# An Electrochemiluminescence Immunosensor Based on Gold-Magnetic Nanoparticles and Phage Displayed Antibodies

**DOI:** 10.3390/s16030308

**Published:** 2016-02-27

**Authors:** Xihui Mu, Zhaoyang Tong, Qibin Huang, Bing Liu, Zhiwei Liu, Lanqun Hao, Hua Dong, Jinping Zhang, Chuan Gao

**Affiliations:** State Key Laboratory of NBC Protection for Civilian, Beijing 102205, China; mxh0511@sohu.com (X.M.); qibin_huang@126.com (Q.H.); lbfhyjy@sohu.com (B.L.); liuzhw07@lzu.edu.cn (Z.L.); hlq70@163.com (L.H.); yizhe2007@126.com (H.D.); zjp337@126.com (J.Z.); g.ch.chuan@263.net (C.G.)

**Keywords:** gold-magnetic nanoparticles, phage displayed antibody, *Staphylococcus* protein A, ECL immunosensor, ricin

## Abstract

Using the multiple advantages of the ultra-highly sensitive electrochemiluminescence (ECL) technique, *Staphylococcus* protein A (SPA) functionalized gold-magnetic nanoparticles and phage displayed antibodies, and using gold-magnetic nanoparticles coated with SPA and coupled with a polyclonal antibody (pcAb) as magnetic capturing probes, and Ru(bpy)_3_^2+^-labeled phage displayed antibody as a specific luminescence probe, this study reports a new way to detect ricin with a highly sensitive and specific ECL immunosensor and amplify specific detection signals. The linear detection range of the sensor was 0.0001~200 µg/L, and the limit of detection (LOD) was 0.0001 µg/L, which is 2500-fold lower than that of the conventional ELISA technique. The gold-magnetic nanoparticles, SPA and Ru(bpy)_3_^2+^-labeled phage displayed antibody displayed different amplifying effects in the ECL immunosensor and can decrease LOD 3-fold, 3-fold and 20-fold, respectively, compared with the ECL immunosensors without one of the three effects. The integrated amplifying effect can decrease the LOD 180-fold. The immunosensor integrates the unique advantages of SPA-coated gold-magnetic nanoparticles that improve the activity of the functionalized capturing probe, and the amplifying effect of the Ru(bpy)_3_^2+^-labeled phage displayed antibodies, so it increases specificity, interference-resistance and decreases LOD. It is proven to be well suited for the analysis of trace amounts of ricin in various environmental samples with high recovery ratios and reproducibility.

## 1. Introduction

Electrochemiluminescence (ECL) immunosensors are used widely in biological detection due to their excellent sensitivity, specificity, stability, low background signals and easy manipulation [1,2]. Conventional ECL immunosensors usually employ a polyclonal antibody (pcAb) or monoclonal antibody (mcAb) labeled by a luminescent molecule to act as a luminescence probe. Because the range of groups which can be labeled on the surface of a polyclonal or monoclonal antibody molecule is limited, and multiple site labeling would lower the binding activity of the antibody and antigen, the sensitivity of conventional ECL immunosensors couldn’t be further improved, so much effort has been made to identify new antibody molecules which have more binding sites for labels. When the amount of labels increases, the antibody molecule maintains the binding activity to the target molecule, and the detection signal can be specifically amplified. As a kind of promising recognition molecule for immunodetection, phage displayed antibodies show broad application prospects. Compared with polyclonal antibodies or monoclonal antibodies, phage displayed antibodies offer high yields, small molecular weight, good stability, high affinity and sharp specificity [3,4,5,6,7]. Phage displayed antibodies show both antigen-binding properties and phage-like structures. A phage displayed antibody contains multiple capsid protein copies (about 2700 copies of pVIII). When a signal probe is constructed with this structural advantage and used in immunoassay, an amplifying effect for the specific signal of a target molecule will result. In [8], Kim and others used a phage displayed antibody labeled by horseradish peroxidase as a specific signal detection probe and achieved the quantitative detection of 3-phenoxybenzoic acid by magnetic immunoassay. Our research group has developed magnetic affinity immunoassays based on phage displayed antibodies labeled by enzymes, and achieved the detection of many kinds of toxin (*β*-bungarotoxin, *Staphylococcus aureus* enterotoxin B and abrin) [9,10,11]. However, because of its high molecular weight (40 kDa), when horseradish peroxidase was labelled, greater spatial hindrance occurs and this reduces the binding activity. Moreover, the enzyme is unstable and easy inactivated, so applications of phage antibodies labeled by enzymes are limited in detection capability. The use of a small molecule label instead of an enzyme to label phage displayed antibodies as reporter molecules, has been the focus of several studies. Ru(bpy)_3_^2+^ is stable and has a small molecular weight. When it is used to label a phage displayed antibody, the spatial hindrance produced is small, and the capsid protein of the phage displayed antibody can carry more such labels. Meanwhile the binding activity of the phage displayed antibody and target molecule are maintained. 

ECL immunosensors with magnetic particles as solid carrier are characterized by rapid separation, easy manipulation and strong anti-interference capability. In recent years, they have been widely applied in the detection of protein molecules such as AFP, anti-P53 antibody, CRP, CEA, *etc.* [12,13,14,15,16,17,18]. As a new functionalized magnetic material, gold-magnetic nanoparticles are inorganic magnetic nanocompounds formed from gold nanoparticle-coated super-paramagnetic Fe_3_O_4_ nanoparticles, which have the double advantages of gold nanoparticles and magnetic nanoparticles. Beyond enrichment and separation, they have characteristic good biocompatibility [19,20,21,22,23,24,25]. *Staphylococcus* protein A (SPA) can be linked with the Fc fragment of IgG molecules, whose Fab fragment are exposed outside, by hydrophobic interactions. This oriented fixation is better organized than direct physical adsorption or covalent binding, and it has less impact on the activity of antibodies [11,26,27,28].

In this study, the authors chose ricin as the target molecule, SPA-coated gold-magnetic nanoparticles coupled with anti-ricin pcAb as the magnetic capturing probe, and a Ru(bpy)_3_^2+^-labeled phage displayed antibody as the specific luminescence probe, thus combining the various advantages of gold-magnetic nanoparticles and Ru(bpy)_3_^2+^-labeled phage displayed antibodies, and established a new ECL immunosensor design with high sensitivity and specificity for ricin detection. Through comparison with other types of ECL immunosensor, we focused on the amplifying effects of gold-magnetic nanoparticles, SPA and the Ru(bpy)_3_^2+^-labeled phage displayed antibodies. In the absence of bioterrorism or food poisoning incidents, it is difficult to obtain the actual material or real samples polluted by ricin. We therefore focused on the detection of simulated samples, using river water, fertilized soil (organic matter content >5%), butter biscuit (fat content >30%) and whole rabbit blood as a matrix that was then spiked with ricin in our lab. This study should provide technical support and a reference for clinical diagnosis, environmental monitoring, food hygiene inspection and anti-bioterrorism applications.

## 2. Materials and Methods

### 2.1. Reagents and Instruments

Ricin standard substance [29], anti-ricin polyclonal antibody [30,31], anti-ricin phage displayed antibody, abrin [9,32], super-paramagnetic Fe_3_O_4_ nanoparticles (magnetic nanoparticles, 15 nm) [11] and gold-magnetic nanoparticles (150 nm) were prepared in our lab. *Staphylococcus aureus* enterotoxin B (SEB), HAuCl_4_·H_2_O, hydroxylamine hydrochloride, FeCl_3_·6H_2_O, FeCl_2_·4H_2_O, polyethylene glycol 6000 (PEG6000), *Staphylococcus* protein A (SPA), bovine serum albumin (BSA), bis (2,2’-bipyridine)-4,4’-dicarboxybipyridine-ruthenium di(N-succinimidyl ester) (Ru(bpy)_3_^2+^-NHS ester), N,N’-dimethylformamide (DMF) and tripropylamine (TPA) were purchased from Sigma (St. Louis, MO, USA). Mouse ScFv Module/Recombinant Phage Antibody System and *E. coli* TG1 were purchased from Amersham Biosciences (Uppsala, Sweden). Horseradish peroxidase conjugated goat anti-mouse IgG (HRP-goat anti-mouse IgG) was purchased from Beijing Biosynthesis Biotechnology Co., Ltd. (Beijing, China). Horseradish peroxidase conjugated anti-M13 monoclonal antibody (HRP-anti-M13 mcAb) was purchased from GE Healthcare Europe GmbH (Freiburg, Germany). The ECL substrate (ProCell) and the cleaning solution (CleanCell) were purchased from Biolot Diagnostics Co., Ltd. (Beijing, China). Filtrate tube of 50,000 molecular weight cut-off (MWCO) (Vivalpin 500 μL) was purchased from Sartorius Stedim Biotech GmbH (Goettingen, Germany). River water was obtained from the Kunyu River (Beijing, China). Fertilized soil (organic matter content >5%) was from the Fenghuang Ridge (Beijing, China), Butter biscuit (fat content >30%) was obtained from Master Kong, Ltd. (Beijing, China). Whole rabbit blood was from venous blood extracted from rabbit. The above four samples were all randomly collected from the environment.

A_280nm_ and A_450nm_ values were respectively determined on a BioMATE 3S UV-Vis spectrophotometer (Thermo Fisher Scientific Inc., Waltham, MA, USA) and a Type 680 microplate reader (Bio-Rad, Hercules, CA, USA). Affinity was determined on ProteOn XPR Protein Interaction Array System (Bio-Rad). Magnetic separation operation was carried out on magnetic separation rack (Dynal Biotech GmbH, Hamburg, Germany). ECL reactions were performed on the immuno-magnetic electrochemiluminescence instrument developed by our laboratory and Xianruimai Analytical Instruments Co., Ltd. (Xi’an, China).

### 2.2. Preparation of Gold-Magnetic Nanoparticles

Gold-magnetic nanoparticles were synthesized according to a literature method [33] with slight modifications. Super-paramagnetic Fe_3_O_4_ nanoparticles (0.2 g) obtained using the microwave co-precipitation method were dissolved in ultrapure water (100 mL), then ultrasonicated for 15 min. Then 1% HAuCl_4_·4H_2_O (10 mL) was added the solution and allowed to react at room temperature for 1 h with a stirring speed of 1500 rpm. Next 50 mM hydroxylamine hydrochloride (40 mL) was added the solution and allowed to react at room temperature for 1 h with a stirring speed of 2000 rpm. Magnetic separation was performed and the supernatant was discarded. 1 M HCl (100 mL) was added and stirred for 4 h to remove the uncoated Fe_3_O_4_ nanoparticles. The gold nanoparticles produced in the preparation were removed by magnetic separation at the same time. The final product was then washed with ultrapure water to neutrality and diluted to 50 mL for later use. The yield of solid product was calculated. 

### 2.3. Preparation and Characterization of Anti-Ricin Phage Displayed Antibody

#### 2.3.1. Preparation of a Large Phage Displayed Single-Chain Fragment Variable (scFv) Antibody Library

The phage displayed single-chain fragment variable (ScFv) antibody library was prepared according to standard protocols [4]. The variable heavy chain gene region (VH) and variable light chain gene (VL) region of the antibody were amplified using a Mouse ScFv Module/Recombinant Phage Antibody System and assembled into the ScFv gene segment, which was combined with vector PHB-1HSCFV, and the vector pHB-mScFv was constructed and transformed into TG1 cells. Transformed bacteria were plated onto a LB plate (containing 170 μg/mL chloramphenicol), and cultivated at 37 °C overnight. The next day, cultured bacteria were suspended in 2×YT medium (containing 50 μg/mL chloramphenicol and 20% glucose), and shake-cultured at 37 °C and 250 rpm until the OD_600_ reached 0.5. M13KO7 helper phage was added to the cultivated bacteria with a ratio of 1:10, and shake-cultured at 37 °C and 150 rpm for 1 h, centrifuged and resuspended in 200 mL 2×YT medium (containing 170 μg/mL chloramphenicol and 50 μg/mL kanamycin), and shake-cultured at 37 °C and 250 rpm overnight. The next day, the cultured bacteria were centrifuged and the supernatant collected. Phage was purified by polyethylene glycol/NaCl precipitation and resuspended in a total volume of 2 mL PBS. Thus the phage displayed antibody library was obtained and the titer was determined.

#### 2.3.2. Screening of Anti-Ricin Phage Displayed Antibody

Anti-ricin phage displayed antibody was synthesized according to the literature method with slight modifications [34,35]. The ricin was diluted to 50 μg/mL with 0.05 M Na_2_CO_3_-NaHCO_3_ buffer (pH 9.6), then was incubated in 96-well microplates at 4 °C overnight for immobilization. Each well was washed three times with PBST (0.01 M PBS buffer, containing 0.05% Tween-20, pH 7.4). Blocking buffer (0.01 M PBS buffer, containing 1% BSA, pH 7.4) was added (150 μL/well) and incubated at 37 °C for 1 h, and the wells were then washed with PBST three times. Phage displayed antibody ScFv library (300 μL) was reacted with 100 μL of blocking buffer at room temperature for 15 min, then added to each well, and incubated at 37 °C for 2 h. The phage displayed antibody ScFv library was extracted, and washed 10 times with PBST and PBS, respectively, 0.1 M glycine-HCl buffer (pH 2.2, 100 μL) was added to each well, reacted at 37 °C for 15 min, then neutralized with 6 μL of 2 M Tris and collected. Eluted phages (10 μL) were added to 5 mL *E. coli* TG1 mid-log cells. The mixture was incubated at 37 °C for 20 min, and then plated onto a LB plate (containing 50 μg/mL chloramphenicol). The amount of phages eluted was calculated. The rest of the eluted phages was added to 5 mL *E. coli* TG1 mid-log cells. The mixture was incubated at 37 °C for 20 min, centrifuged and resuspended in a total volume of 1 mL 2×YT medium, then plated onto a LB plate (containing 170 μg/mL chloramphenicol), at 37 °C overnight. The next day, bacterial colonies on the plate were suspended, rescued with M13KO7 helper phage and extracted, the phage antibody titer was determined, and then the recovery ratio can be calculated. For subsequent phage selections, the steps were same as in the first round, however the concentration of ricin for immobilization was gradually decreased, to 25 μg/mL in the second round and 15 μg/mL in the third round.

#### 2.3.3. Characterization of Anti-Ricin Phage Displayed Antibody Positive Clones by ELISA

From the plate which was used to determine the titer of phage antibody after three rounds, 20 bacterial colonies were selected to add to 5 mL 2×YT medium (containing 170 μg/mL chloramphenicol), grown until the OD_600_ reached to 0.5. M13KO7 helper phage was added, incubated at 37 °C and 150 rpm for 1 h, centrifuged and resuspended in 5 mL 2×YT medium (containing 170 μg/mL chloramphenicol and 50 μg/mL kanamycin), incubated at 30 °C and 250 rpm overnight. The next day, bacterial solution was centrifuged and the supernatant obtained. Selected phage displayed antibody-positive clones by indirect ELISA.

#### 2.3.4. DNA Sequencing and Binding Affinity of Anti-Ricin Phage Displayed Antibody

DNA sequencing was conducted with selected phage displayed antibody positive clones. The binding affinity of anti-ricin phage displayed antibody and ricin was determined by a PlexArray^TM^ Kx5 System.

### 2.4. Preparation of the SPA-Coated Gold-Magnetic Nanoparticles Functionalized Capturing Probes

Gold-magnetic nanoparticles (1 mg) were washed with 0.01 M PBS buffer (pH 7.4) and magnetic separation was used to discard the supernatant. Then a certain amount of 1 mg/mL SPA was added. By physical adsorption, SPA was immobilized on the surface of the gold-magnetic nanoparticles, and reacted at room temperature for 2 h with stirring, followed by washing with 0.01 M PBS buffer (pH 7.4) and magnetic separation to remove the supernatant. A certain amount of 1 mg/mL ricin polyclonal antibody was added to allow to react at room temperature for 1 h with stirring, the gold-magnetic nanoparticles were then washed with 0.01 M PBS buffer (pH 7.4), diluted to 1 mL and stored at 4 °C until use. The final concentration was 1 mg/mL. 

### 2.5. Preparation of ECL Probes

The authors adopted the method of [9] to prepare ECL probes. Anti-ricin phage displayed antibody (100 μL) with a titer of 1 × 10^14^ PFU/mL (1.6 × 10^−7^ M) and 500 μL of 0.05 M carbonate buffer (pH 9.6) were mixed. Then 300 μL of Ru(bpy)_3_^2+^-NHS ester with concentration of 1 mg/mL (8.76 × 10^−4^ M) and DMF were added and allowed to react at room temperature and away from light for 12 h with stirring. Unbound Ru(bpy)_3_^2+^-NHS ester was removed by filtrate tube, and the ECL probes were resuspended in 0.01 M PBS buffer (pH 7.4) to 1 mL and stored at 4 °C and away from light until use. The final concentration was 1.6 × 10^−^^8^ M.

### 2.6. Establishment of the ECL Immunosensor Based on Gold-Magnetic Nanoparticles and Phage Displayed Antibody

**Figure 1:**
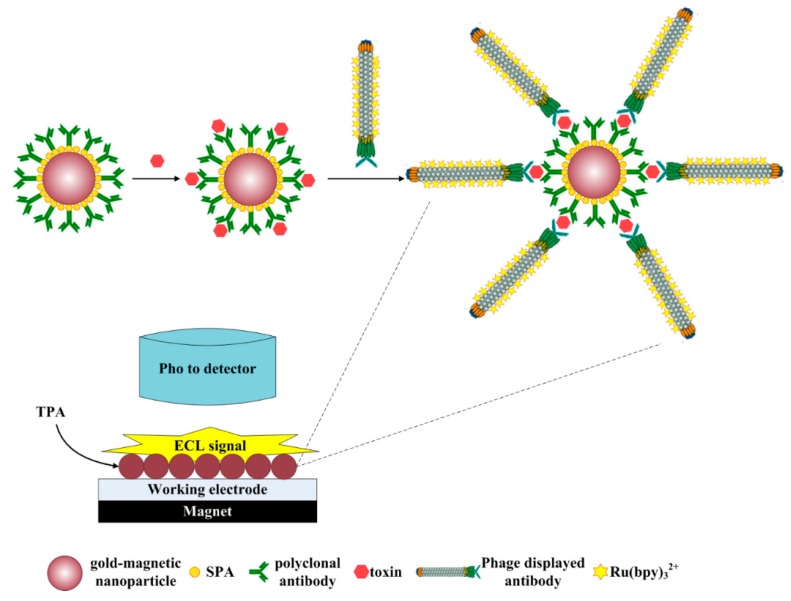
Model of detection toxin by ECL immunosensor based on gold-magnetic nanoparticles and phage displayed antibody.

The 1 mg/mL SPA-coated gold-magnetic nanoparticles coupled with polyclonal antibody capturing probe (100 μL) and simulated samples or ricin standard substances of different concentrations (100 μL), which were diluted with dilution buffer (0.01 M PBS buffer, pH 7.4), were mixed at room temperature for 15 min with stirring, then washed two times with 0.01 M PBS buffer (pH 7.4) and diluted to 200 μL. Then 100 μL of 1.6 × 10^−^^8^ M ECL probe was added and mixed at room temperature for 1 h with stirring, washed two times with 0.01 M PBS buffer (pH 7.4) and diluted to 200 μL to obtained the gold-magnetic nanoparticles complex. The gold-magnetic nanoparticles complex (20 μL) was injected into the detector cell of the ECL immunosensor, and deposited on the surface of the working electrode by the action of a magnet. Then, by setting the voltage between the working electrode and the reference electrode at 1.25 V, the ECL reaction between Ru(bpy)_3_^2+^-labeled phage displayed antibody and tripropylamine (TPA) in the solution was triggered, generating photons. The optical signal was detected by a photomultiplier tube (PMT) detector [36]. The strategy is shown in Figure 1. 

### 2.7. Regeneration of the Electrodes Surface of ECL Immunosensor

After the tests, the magnet on the electrode was removed. The detector cell of the ECL immunosensor was repeatedly washed with ultrapure water and cleaning solution (CleanCell) was injected. On the electrodes surface a step pulse reaction occurred, then the electrodes were washed with ultrapure water and ECL substrate (ProCell) until the ECL intensity value recovered its baseline level and can be used again. 

### 2.8. Limit of Detection, Linear Range and Specificity

Based on the calibration curve of the ECL immunosensor (method 1), limit of detection, linear range and other parameters of this method were determined, and 1 μg/L abrin, SEB, BSA and other non-target proteins were also analyzed using this method. In order to examine the specificity of this method, the results were compared with those of ricin. Under the same conditions, five ECL immunosensors were compared, namely SPA-coated gold-magnetic nanoparticles coupled with polyclonal antibody capturing probe-toxins-Ru(bpy)_3_^2+^-labeled phage displayed antibody luminescence probe detection scheme (Method 1), SPA-coated magnetic nanoparticles coupled with polyclonal antibody capturing probe-toxins-Ru(bpy)_3_^2+^-labeled phage displayed antibody luminescence probe detection scheme (Method 2), gold-magnetic nanoparticles coupled with polyclonal antibody capturing probe-toxins-Ru(bpy)_3_^2+^-labeled phage displayed antibody luminescence probe detection scheme (Method 3), SPA-coated gold-magnetic nanoparticles coupled with polyclonal antibody capturing probe-toxins-Ru(bpy)_3_^2+^-labeled monoclonal antibody luminescence probe detection scheme (Method 4) and magnetic nanoparticles coupled with polyclonal antibody capturing probe-toxins-Ru(bpy)_3_^2+^-labeled mcAb luminescence probe detection scheme (Method 5). Through comparison of the five ECL immunosensors, the signal amplifying effects of gold-magnetic nanoparticles, SPA-oriented antibody and Ru(bpy)_3_^2+^-labeled phage displayed antibody were investigated. In addition, through comparison with conventional double-antibody sandwich ELISA (Method 6), the low LOD performance of the ECL immunosensor based on gold-magnetic nanoparticles and Ru(bpy)_3_^2+^-labeled phage displayed antibody was investigated.

### 2.9. Measurement of Simulated Ricin Samples

Fertilized soils (1 g, organic matter >5%), butter biscuit (1 g, fat content >30%), whole rabbit blood (10 μL) and river water (1 mL) were added into 4 μL of 10 mg/L ricin standard. Then the mixture was diluted to 8 mL with dilution buffer (0.01 M PBS buffer, pH 7.4) to get a final ricin concentration of 5 μg/L. The river water samples were directly measured, and the fertilized soils samples were centrifuged at 5000 g for 20 min, while butter biscuit and whole rabbit blood samples were centrifuged at 10,000 g for 15 min and 10 min, respectively. The supernatants were then collected and the recovery rate, relative standard deviation and other indexes of the detection were analyzed and calculated.

## 3. Results

### 3.1. Preparation of Gold-Magnetic Nanoparticles

**Figure 2:**
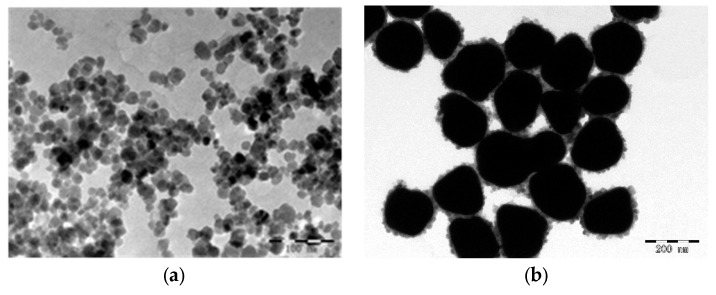
TEM images of magnetic nanoparticles (**a**) and gold-magnetic nanoparticles (**b**).

By combining microwave treatment with conventional chemical co-precipitation procedures, our research group obtained super-paramagnetic Fe_3_O_4_ nanoparticles with excellent dispersion, spherical morphology, a particle size of 15 nm, and a saturation magnetization of 78.875 emu/g. Compared with conventional co-precipitation methods, this method simplifies the preparation of Fe_3_O_4_ magnetic nanoparticles and shortens the reaction time. Based on this our research group obtained gold-magnetic nanoparticles with excellent dispersion, spherical morphology, particle size of 150 nm, saturation magnetization of 63.151 emu/g and a solid yield of 2 mg/mL using the hydroxylamine hydrochloride reduction method. Figure 2 shows a TEM image of the magnetic nanoparticles and gold-magnetic nanoparticles. The yields of solid magnetic nanoparticle and gold-magnetic nanoparticle products were 6 mg/mL and 2 mg/mL, respectively.

### 3.2. Preparation of Magnetic Ricin-Capturing Probe

#### 3.2.1. Optimal Amount of Immobilized Anti-Ricin Polyclonal Antibody

**Figure 3:**
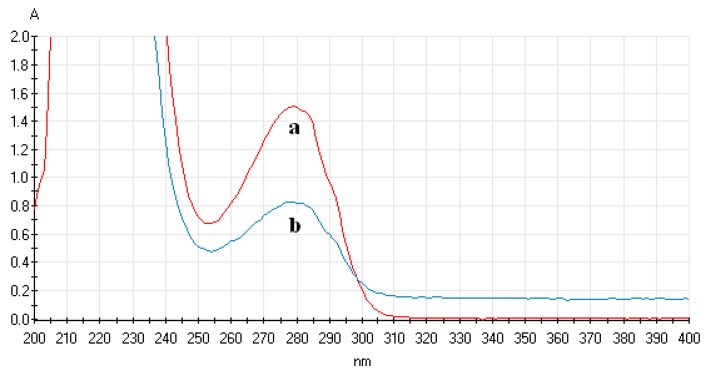
UV-Vis spectrum of SPA solution before and after binding to magnetic nanoparticles ((**a**) UV-Vis spectrum of 240 μg of SPA solution before binding to magnetic nanoparticles; (**b**) UV-Vis spectrum of 240 μg of SPA solution after binding to magnetic nanoparticles).

**Figure 4:**
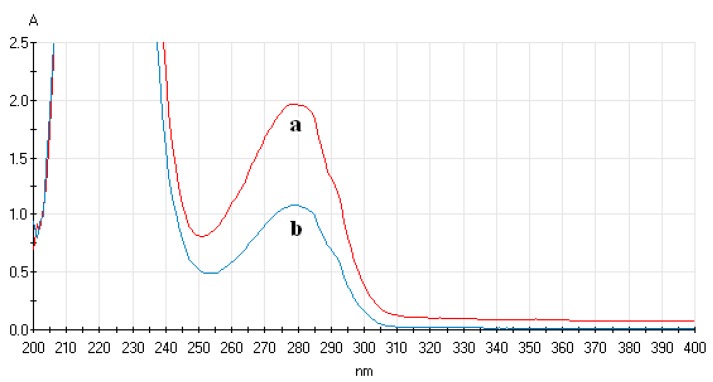
UV-Vis spectrum of SPA solution before and after binding to gold-magnetic nanoparticles. (**a**) UV-Vis spectrum of 320 μg of SPA solution before binding to gold-magnetic nanoparticles; (**b**) UV-Vis spectrum of 320 μg of SPA solution after binding to gold-magnetic nanoparticles).

First, by physical adsorption of magnetic nanoparticles or gold-magnetic nanoparticles and protein, a certain amount of SPA was immobilized on the surface of the magnetic nanoparticles and gold-magnetic nanoparticles. A_280nm_ of the SPA solution was determined before and after immobilization, and the amount of immobilized SPA per mg of magnetic nanoparticles and gold-magnetic nanoparticles was calculated. As shown in Table 1 and Table 2, as the amount of added SPA was increased, the amount of immobilized SPA binding to the magnetic nanoparticles and gold-magnetic nanoparticles gradually increased and tended to reach saturation. It was confirmed that the optimal amount of immobilized SPA on 1 mg of magnetic nanoparticles and gold-magnetic nanoparticles was 240 µg and 320 µg, respectively, and the amount of immobilized SPA on 1 mg of magnetic nanoparticles and gold-magnetic nanoparticles was 111 µg and 168 µg, respectively. Figure 3 shows the UV-Vis spectrum of 240 μg SPA solution before and after binding to magnetic nanoparticles. Figure 4 shows the UV-Vis spectrum of 320 μg SPA solution before and after binding to gold-magnetic nanoparticles.

**Figure 5:**
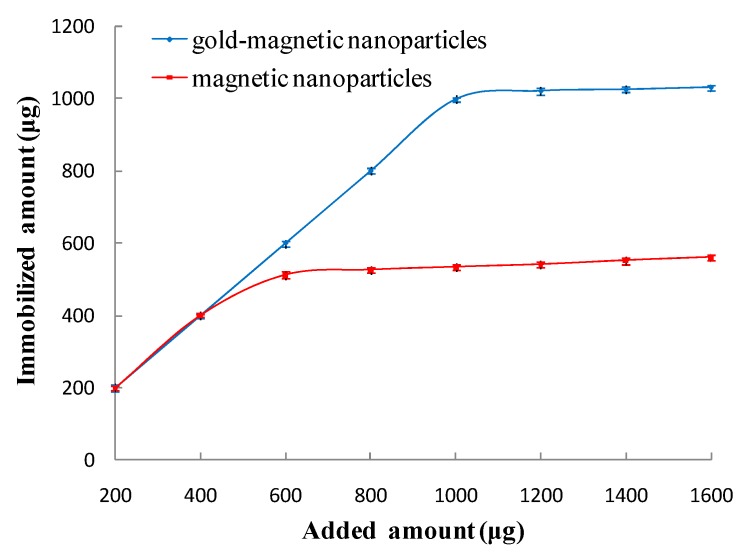
Immobilization ability of polyclonal antibody on 1 mg of gold-magnetic nanoparticles and magnetic nanoparticles.

Since SPA was specifically bound to the Fc fragment of IgG molecules, a certain amount of anti- ricin polyclonal antibody (pcAb) was added to get the SPA-coated magnetic nanoparticle functionalized capturing probe and SPA-coated gold-magnetic nanoparticle functionalized capturing probe. A_280__nm_ of the anti-ricin pcAb solution was determined before and after immobilization, and according to the results, the actual amount of immobilized anti-ricin pcAb on 1 mg of magnetic nanoparticles and gold-magnetic nanoparticles was calculated to be 512 µg and 998 µg, respectively (Figure 5). Theoretically, the amount of immobilized anti-ricin pcAb to 1 mg of magnetic nanoparticles and gold-magnetic nanoparticles respectively was 845 µg and 1280 µg as calculated according to the amount of immobilized SPA. The actual amount was 60.6% and 77.9% of the theoretical maximum. SPA is bivalent, which means that each SPA molecule can theoretically bind 2 IgG molecules. However, due to the steric hindrance effect, each SPA molecule bond only 1.2 molecules and 1.6 molecules of anti-ricin pcAb molecules in practice. 

Compared with the SPA-coated magnetic nanoparticles functionalized capturing probe, the SPA-coated gold-magnetic nanoparticles functionalized capturing probe has more amount of immobilized SPA and anti-ricin pcAb, indicating that the gold-magnetic nanoparticles can provide more active surface and more biomolecule probe immobilization. In addition SPA can accomplish orientation immobilization and provide an ordered arrangement of antibody on surface of gold-magnetic nanoparticles, the steric hindrance effect of the molecules is reduced, thus the activity of the functionalized capturing probe was improved and and detection limit was decreased.

To verify that the oriented immobilization of antibody occurred via the use of *Staphylococcus* protein A rather than of physical adsorption, a control experiment was performed. First, a certain amount of BSA was immobilized on the surface of the gold-magnetic nanoparticles by physical adsorption. A_280nm_ of the BSA solution was determined before and after immobilization, and the amount of immobilized BSA per mg of gold-magnetic nanoparticles was calculated. As shown in Table 3, as the amount of added BSA was increased, the amount of immobilized BSA binding to gold-magnetic nanoparticles gradually increased and tended to reach saturation. It was confirmed that the optimal amount of added BSA for 1 mg of gold-magnetic nanoparticles was 400 µg, and the amount of immobilized BSA on 1 mg of gold-magnetic nanoparticles was 265 µg.

**Figure 6:**
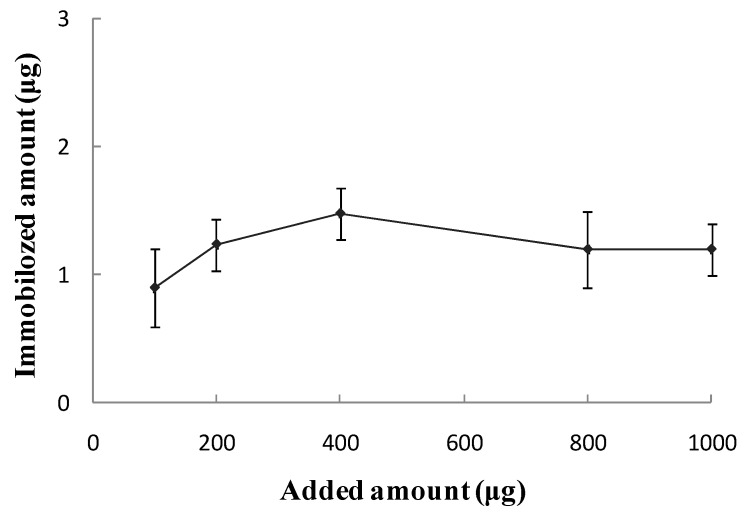
Immobilization ability of polyclonal antibody on 1 mg of gold-magnetic nanoparticles.

Then a certain amount of anti- ricin pcAb was added. A_280__nm_ of the anti-ricin pcAb solution was determined before and after immobilization, and the actual amount of immobilized the anti-ricin pcAb was calculated. As shown in Figure 6, as the amount of added anti-ricin pcAb was increased, the amount of immobilized anti-ricin pcAb remained at 0.9 μg~1.48 μg and no longer increased. It was shown that the sites on the surface of the gold-magnetic nanoparticles were occupied by BSA, and the anti-ricin pcAb couldn’t be immobilized on the surface of the gold-magnetic nanoparticles any more by physical adsorption, so the oriented immobilization of anti-ricin pcAb occurred via the use not of physical adsorption but of *Staphylococcus* protein A.

#### 3.2.2. Qualification of the SPA-Coated Gold-Magnetic Nanoparticles Functionalized Capturing Probe

##### The Magnetic Features of the SPA-Coated Gold-Magnetic Nanoparticles Functionalized Capturing Probe

**Figure 7:**
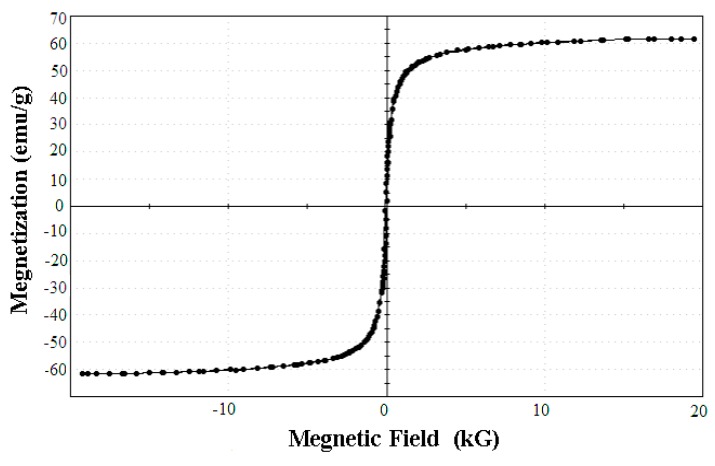
Magnetic hysteresis loops curve of the SPA-coated gold-magnetic nanoparticles functionalized capturing probe.

The magnetic features of the SPA-coated gold-magnetic nanoparticles functionalized capturing probe was checked by VSM (see Figure 7), and the saturation magnetization of the magnetic ricin-capturing probe was 61.357 emu/g. Compared with gold-magnetic nanoparticles, their saturation magnetization could be considered the same. The result showed that prepared the SPA-coated gold-magnetic nanoparticles functionalized capturing probe had good magnetic features.

##### Biological Activity of the SPA-Coated Gold-Magnetic Nanoparticles Functionalized Capturing Probe

**Figure 8:**
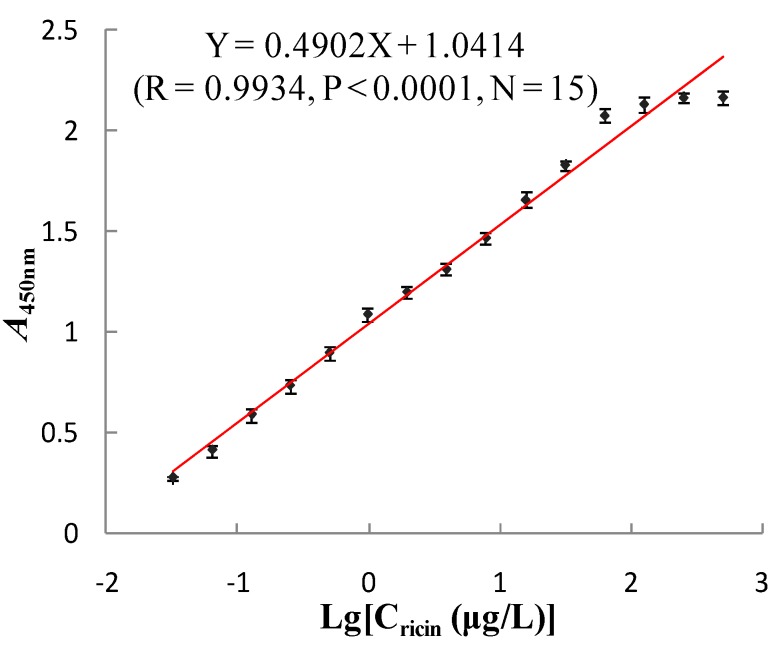
Determination activity of the SPA-coated gold-magnetic nanoparticles functionalized capturing probe.

Biological activity of the SPA-coated gold-magnetic nanoparticles functionalized capturing probe was validated. The SPA-coated gold-magnetic nanoparticles functionalized capturing probe (100 μL) was mixed with 100 μL of ricin (0.032~500 μg/L) to react. Then anti-ricin mcAb (100 μL, 1 mg/mL) and HRP-goat anti-mouse IgG (100 μL) were added. Under the same conditions, double-antibody magnetic affinity immunoassay (MAIA) was used to determine the A_450nm_. According to the change of the A_450nm_, the biological activity of the gold-magnetic nanoparticles functionalized capturing probe was checked. As shown in Figure 8, as the concentration of ricin increased, A_450nm_ gradually increased as well, indicating that the probe can combine with the target toxin with a good dose-effect relationship. The logarithm of ricin concentration and A_450nm_ showed a significant rectilinear correlation, and the regression equation was Y = 0.4902X + 1.0414 (*R* = 0.9934, *P* < 0.0001, *N* = 15). The results showed that prepared the SPA-coated gold-magnetic nanoparticles functionalized capturing probe with good activity can be used in the test.

### 3.3. The Preparation and Characterization of Anti-Ricin Phage Displayed Antibody

#### 3.3.1. Screening of Anti-Ricin Phage Displayed Antibody 

After amplification of the phage displayed ScFv antibody library (titer of 1.1 × 10^13^ PFU/mL), affinity panning was processed by “adsorption-elution-amplification” for three rounds, with ricin as target molecule. In the process, the amount of ricin was reduced step by step to improve the panning effects. In the first round, the amount of coated ricin on a 96-well microplate was 5 μg per well. The amount was 2.5 μg per well in the second round and 1.5 μg per well in the third round, respectively. In each round, the titer of eluted phage was determined to calculate the recovery ratio. As shown in Table 4, the titer of eluted phage and recovery ratio both increased in the process, the phage which carried anti-ricin antibody enriched. After third round, the recovery ratio was not significantly increased any more, and the screening ended.

#### 3.3.2. Characterization of Anti-Ricin Phage Displayed Antibody Positive Clones by ELISA

**Figure 9:**
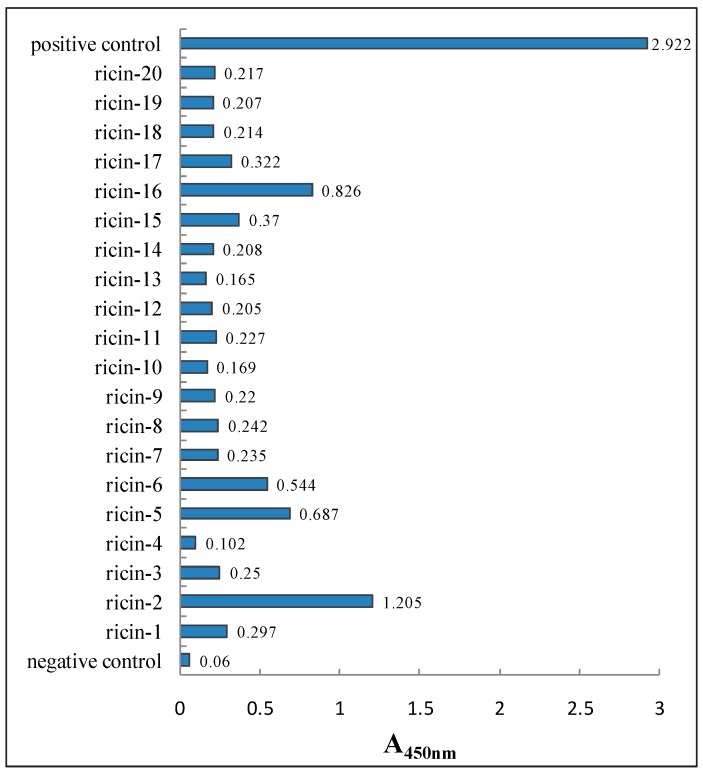
Determination of absorbance values of the 20 anti-ricin phage displayed antibody clones by ELISA.

After affinity panning for three rounds, 20 anti-ricin phage displayed antibody clones were chosen randomly to be detected by indirect ELISA. At the same time, BSA and M13KO7 helper phage were detected as a negative control and positive control, respectively. P/N > 2.1 was deemed positive. The results were shown in Figure 9. No. 2, No. 5 and No. 15 clone showed higher A_450nm_. With the highest A_450nm_, No. 2 clone was chosen to perform DNA sequencing and affinity determination. 

#### 3.3.3. DNA Sequencing and Binding Affinity of Anti-Ricin Phage Displayed Antibody

Plasmid was extracted from No. 2 clone positive clone and DNA sequencing was conducted; the sequence of the complete assembled ScFv antibody was as follows:

5’-GGCCCAGCCGGCC$\underset{=}{\text{ATG}}$GCACAAGTTCAGCTGGTTGAATCTGGTGCAGAAGTTGTTAAACCGGGTGCTTCCGTTAAAATGTCTTGCAAAGCATCCGGTTACACTTTCACCTCCTACCCGATCGAATGGATGAAACAGGCTCCGGGTAAATCCCTGGAGTGGATCGGTAACTTTCACCCGTACAACGACGACACTAAGTACAACGAGAAGTTCAAAGGTCGTGCTACTCTGACTGTTGACACTTCTACCTCTACTGTTTACCTGGAACTGTCCTCTCTGCGTTCTGAAGATACTGCTGTTTACTACTGCGCTATCTACTTCGGTAAGCCGTGGTTCACTTACTGGGGTCAGGGTACTCTGGTTACTGTTTCTTCCGGTGGAGGCGGTTCAGGCGGAGGTGGCTCTGGCGGTGGCGGATCGGAAATCGAACTGACCCAGTCTCCGGGTACCATGTCTGCTTCTCCGGGTGAACGTGTTACTATGACTTGCCGTGCTTCTTCCTCTGTTTCTTCCAACTACCTGCACTGGTATCAGCAAAAACCGGGTCAGTCTCCGAAGCCGTGGATCTACGGTACTTCTAACCTGGCTTCTGGTGTTCCGGACCGTTTCTCTGGTAGCGGTTCTGGTACTTCCTACTCTCTGACTATCACTTCTATGGAACCGGAAGACGCTGCTACTTACTACTGCCAGCTGTGGAACTACCCGCTGTACACTTTCGGTGGTGGTACCAAACTGGAGATCAAACGCGCGGCCGC-3’.

In this DNA sequencing, the single underlined part “GGCCCAGCCGGCC” and “GCGGCCGC” respectively represents the restriction enzyme site of Sfi I(5’) and Not I(3’), and the double underlined part “ATG” represents the initiation codon. The ORF contained 732 bases, from which 244 amino acids were encoded. Binding affinity of anti-ricin phage displayed antibody to ricin was determined using a PlexArray™ Kx5 System. The determined Ka of anti-ricin phage displayed antibody was 1.48 × 10^9^ M^−1^, and the determined Kd of anti-ricin phage displayed antibody was 6.75 × 10^−10^ M. The binding affinity between anti-ricin phage displayed antibody and ricin was shown to be strong. 

### 3.4. The Qualification Feature of ECL Probe

#### 3.4.1. Analysis of UV-Vis Spectrum of ECL Probe

**Figure 10:**
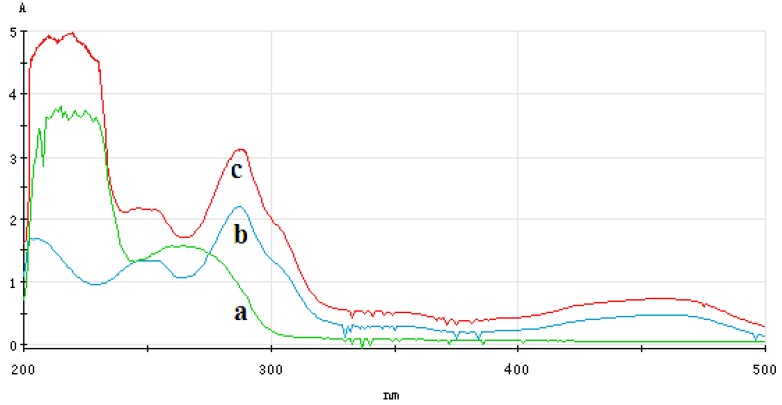
UV-Vis spectrum of ECL probe ((**a**) UV-Vis spectrum of phage displayed antibody; (**b**) UV-Vis spectrum of Ru(bpy)_3_^2+^-NHS ester; (**c**) UV-Vis spectrum of Ru(bpy)_3_^2+^-labeled phage displayed antibody).

Figure 10 shows the UV-Vis spectrum of phage displayed antibody solution before and after addition of the Ru(bpy)_3_^2+^ labeled phage displayed antibody ECL probe. In this figure, curve a is the UV-Vis spectrum of phage displayed antibody and the corresponding wavelength of its characteristic absorption peak was 260 nm. Curve b is the UV-Vis spectrum of Ru(bpy)_3_^2+^-NHS ester and the corresponding wavelength of its three characteristic absorption peaks were 247 nm, 287 nm and 458 nm, respectively . Curve c is the UV-Vis spectrum of Ru(bpy)_3_^2+^-labeled phage displayed antibody and the corresponding wavelength of its three characteristic absorption peaks were 247 nm, 288 nm and 458 nm, respectively. The result shows that Ru(bpy)_3_^2+^ has good binding to the anti-ricin phage displayed antibody.

#### 3.4.2. Qualification of the ECL Profile of the ECL Probe

**Figure 11:**
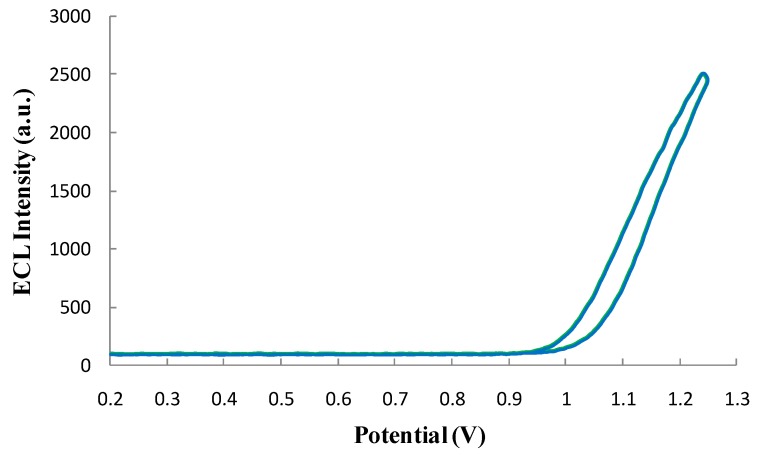
ECL profile of luminescence probe.

The ECL reaction luminescence probe was scanned(cyclic voltammetry method). As shown in Figure 11, the Ru(bpy)_3_^2+^-labeled phage displayed antibody exhibited a strong ECL signal. The maximum ECL intensity (peak potential) was still at 1.25 V, indicating that the luminescence probe maintained the ECL profile of connatural Ru(bpy)_3_^2+^ with a good ECL profile that can meet the demands of ECL reactions.

### 3.5. The Performance of ECL Immunosensor

#### 3.5.1. Linear Range and Limit of Detection

**Figure 12:**
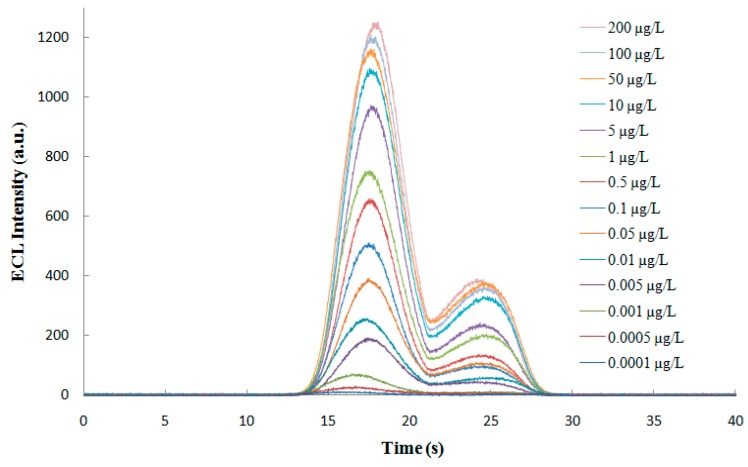
ECL spectra for the ricin detection at different concentrations.

**Figure 13:**
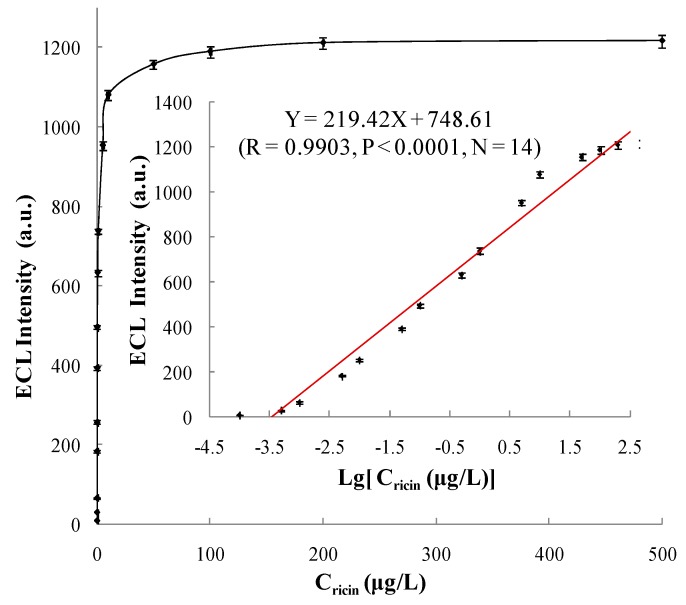
Standard curve of ricin determined by ECL immunosensor.

The established ECL immunosensor was used to test gold-magnetic nanoparticles complex binding of different concentrations of ricin standard substance and obtain the corresponding ECL intensity values. Figure 12 shows the ECL spectra for the ricin detection at different concentrations. Each concentration was tested five times to obtain as average ECL intensity value and deduct the baseline value. As shown in Figure 13, as the concentration of ricin increased, the ECL intensity values increased gradually and tended to reach saturation. When the concentration of ricin was between 0.0001 and 200 µg/L, the logarithm of the ricin concentration and ECL intensity values showed a significant rectilinear correlation, and the regression equation was Y = 219.42X + 748.61 (*R* = 0.9903, *P* < 0.0001, *N* = 14). When the concentration of ricin was 0.0001 μg/L, the “signal-to-noise ratio (S/N)” of the ECL immunosensor was 3 and we took this concentration as the limit of detection (LOD).

**Figure 14:**
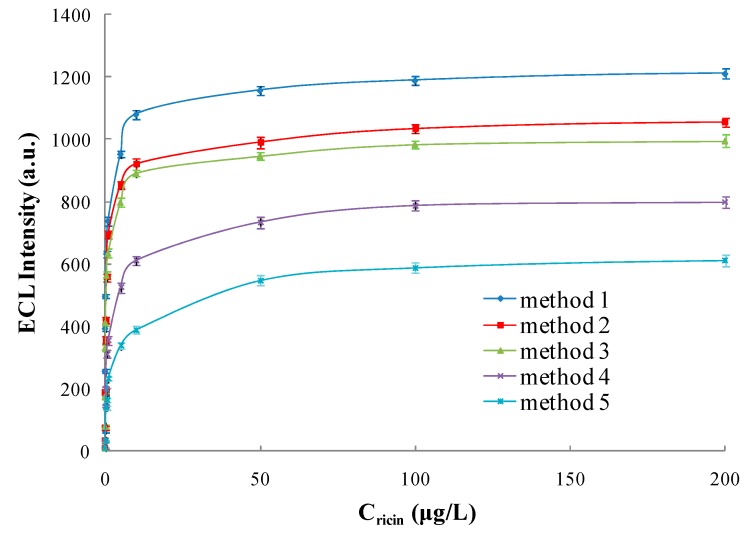
Comparison of five ECL immunosensors (Method 1: SPA-coated gold-magnetic nanoparticles coupled with pcAb capturing probe–toxins–Ru(bpy)_3_^2+^-labeled phage displayed antibody luminescence probe detection scheme; Method 2: SPA-coated magnetic nanoparticles coupled with pcAb capturing probe–toxins–Ru(bpy)_3_^2+^-labeled phage displayed antibody luminescence probe detection scheme; Method 3: gold-magnetic nanoparticles coupled with pcAb capturing probe–toxins–Ru(bpy)_3_^2+^-labeled phage displayed antibody luminescence probe detection scheme; Method 4: SPA-coated gold-magnetic nanoparticles coupled with pcAb capturing probe–toxins–Ru(bpy)_3_^2+^-labeled mcAb luminescence probe detection scheme; Method 5: magnetic nanoparticles coupled with pcAb capturing probe–toxins–Ru(bpy)_3_^2+^-labeled mcAb luminescence probe detection scheme).

Five ECL immunosensors and ELISA were compared, namely SPA-coated gold-magnetic nanoparticles coupled with pcAb capturing probe-toxins-Ru(bpy)_3_^2+^-labeled phage displayed antibody luminescence probe detection scheme (Method 1), SPA-coated magnetic nanoparticles coupled with pcAb capturing probe-toxins-Ru(bpy)_3_^2+^-labeled phage displayed antibody luminescence probe detection scheme (Method 2), gold-magnetic nanoparticles coupled with pcAb capturing probe-toxins-Ru(bpy)_3_^2+^-labeled phage displayed antibody luminescence probe detection scheme (Method 3), SPA-coated gold-magnetic nanoparticles coupled with pcAb capturing probe-toxins-Ru(bpy)_3_^2+^-labeled mcAb luminescence probe detection scheme (Method 4), magnetic nanoparticles coupled with pcAb capturing probe-toxins-Ru(bpy)_3_^2+^-labeled mcAb luminescence probe detection scheme (Method 5) and conventional double-antibody sandwich ELISA (Method 6). As shown in Figure 14 and Table 5, gold-magnetic nanoparticles can decrease LOD 3-fold by comparison between Method 1 and Method 2, SPA can decrease LOD 3-fold according to the comparison between Method 1 and Method 3, Ru(bpy)_3_^2+^-labeled phage displayed antibody can decrease LOD 20-fold from the comparison between Method 1 and Method 4. The ECL immunosensor based on gold-magnetic nanoparticles and phage displayed antibody integrated the three amplifying effects above, and can decrease LOD 180-fold from the comparison between Method 1 and Method 5. Compared with a conventional double-antibody sandwich ELISA assay (Method 6), the detection limit of the ECL immunosensor based on gold-magnetic nanoparticles and phage displayed antibody was decreased 2500-fold.

From the comparison between Method 1 and Method 2, it was proved that the SPA-coated gold-magnetic nanoparticles functionalized capturing probe has a better binding activity than the SPA-coated magnetic nanoparticles functionalized capturing probe. From a comparison between Method 4 and Method 5, it was proved that gold-magnetic nanoparticles have better biocompatibility and can provide more active surface area and more immobilized biomolecule probe, and that SPA can orient and orderly arrange antibodies on gold-magnetic nanoparticles, improving the activity of the gold-magnetic nanoparticles functionalized capturing probe. The amplifying effects of Ru(bpy)_3_^2+^-labeled phage displayed antibody and gold-magnetic nanoparticles functionalized capturing probe in the ECL immunosensor amplified the target signal and decreased the limit of detection greatly. 

So far various technologies have been developed for ricin detection (see Table 6). Some of them, including enzyme-linked immunosorbent assay (ELISA) [37,38], lateral flow devices with electroluminescence detection [39], immunochromatography assay [40], electrochemiluminescence assay [41], magnetoelastic biosensors [42], microring resonator arrays [43], surface plasmon resonance biosensors [44,45], liquid-crystal based sensor [46], electrochemical biosensors [47], microelectrode array biosensors [48], Raman scattering technique [49], immuno-polymerase chain reaction assay [50,51], mass spectrometry [52], *etc.*, are sensitive and rapid. In most of the above technologies, conventional antibodies were used as recognition molecules. The detection of ricin with phage displayed single domain antibodies (sdAb) as the recognition molecule was seldom reported. Using multiple copies of capsid proteins of phage displayed antibody, Goldman [38] established ELISA and Luminex fluid array assays based on phage displayed sdAb, which realized an amplification effect of the specific signal of target molecules with a LOD of 1 ng/mL and 64 pg/mL. In this study, we set up an ECL immunosensor integrating the multiple advantages of an ultra-highly sensitive ECL technique, SPA-coated gold-magnetic nanoparticles which improved the activity of the capturing probe, and the amplifying effect of Ru(bpy)_3_^2+^-labeled phage displayed antibodies. This method decreased the LOD 10,000 times and 640 times, respectively, compared with Goldman’s reported work.

#### 3.5.2. Accuracy and Specificity

Within the linear concentration range, 0.0005, 0.001, 0.01, 0.1, 1, 10 and 100 μg/L of ricin standard substance was tested using our ECL immunosensor. Each concentration was tested five times to obtain the ECL intensity values, which were 30 ± 2, 66 ± 5, 256 ± 5, 496 ± 6, 738 ± 14, 1080 ± 15 and 1189 ± 16 with RSDs of 8.0%, 7.3%, 2.1%, 1.3%, 1.8%, 1.4% and 1.4%, respectively, showing good accuracy.

This ECL immunosensor was also tested with 1 µg/L ricin, abrin, SEB and BSA (Table 7) whose matrix was dilution buffer (0.01 M PBS buffer, pH 7.4), and the negative control was the simulated sample without ricin, for example, dilution buffer, river water, fertilized soil (organic matter content >5%), butter biscuit (fat content >30%) and whole rabbit blood, which separately acted as matrix. The ECL intensity values of the non-target proteins, abrin, SEB, and BSA were close to negative results, indicating the high specificity of this method for the detection of ricin.

#### 3.5.3. Detection of Ricin in Simulated Samples

As shown in Table 8, all simulated samples which contained ricin in river water, fertilized soil (organic matter content >5%), butter biscuit (fat content >30%) and whole rabbit blood were examined using the ECL immunosensor, showing recovery ratios of 90.8%~94.2%.

With the conventional double-antibody sandwich ELISA, experiments were repeated, and the concentration of simulated samples was 5 μg/L. As shown in Table 9, the recovery ratio of ricin in river water and butter biscuit was 92.2% and 91.0%, respectively, and it was above 110% both in fertilized soil and whole rabbit blood. It was absorbance value that was determined using ELISA, and the color of solutions will affect the result of the determination. Because of the colors of fertilized soil and whole rabbit blood solution, the absorbance value of simulated samples was above the actual value, and the recovery ratio increased. However, with the enrichment and separation properties, the ECL immunosensor can overcome the interference of complex backgrounds and separate the samples selectively. This method met the requirements for analysis of the simulated samples above, showing high recovery ratios and reproducibility.

## 4. Discussion

As a new functionalized magnetic material, gold-magnetic nanoparticles have the double advantages of gold nanoparticles and magnetic nanoparticles. Due to their excellent dispersion, stability, biocompatibility, sensitivity, specificity, interference-resistance, and easy manipulation, gold-magnetic nanoparticles have shown good prospects in biomedicine, molecular biology, immunology, cell biology and environmental engineering. In this study, gold-magnetic nanoparticles were used as a magnetic capturing probe carrier, SPA was used to orient and orderly arrange antibodies, and SPA functionalized gold-magnetic nanoparticles were obtained, which have the multiple advantages of gold and magnetic nanoparticles and oriented immobilization of antibodies.

The gold-magnetic nanoparticles were coated with SPA and coupled with pcAb to construct the SPA-coated gold-magnetic nanoparticles functionalized capturing probe. This kind of probe has a better the binding activity, and decreased LOD 3-fold and 9-fold compared with the SPA-coated magnetic nanoparticles functionalized capturing probe and the conventional magnetic nanoparticles functionalized capturing probe. The advantages of SPA-coated gold-magnetic nanoparticles functionalized capturing probe can be summarized as follows: on the one hand, compared with magnetic nanoparticles, the gold-magnetic nanoparticles show significantly more surface area and volume effects, better stability and biocompatibility, and can provide more active surface area and more immobilized biomolecule probe. On the other hand, the SPA can be linked with the Fc fragment of IgG molecules by hydrophobic interactions, so antibody molecule probes were oriented and orderly arranged on the gold-magnetic nanoparticles’ surfaces. This oriented fixation is better organized than either direct physical adsorption or covalent binding, and it has less impact on the activity of antibodies, so the activity of the functionalized capturing probe was improved. In addition, the phage displayed antibody contains multiple capsid protein copies. When the Ru(bpy)_3_^2+^-labeled phage displayed antibody was taken as the luminescence probe, it can be bound with more Ru(bpy)_3_^2+^, compared with conventional Ru(bpy)_3_^2+^-labeled mcAb, so the signal of target molecules can be amplified greatly. In a previous study, our research group has established a magnetic immunoassay and an ECL immunosensor based on phage displayed antibodies [9,10,11]. In this study, we set up an ECL immunosensor integrating the multiple advantages of the ultra-highly sensitive ECL technique, SPA-coated gold-magnetic nanoparticles improving the activity of the capturing probe, and the amplifying effect of Ru(bpy)_3_^2+^-labeled phage displayed antibodies. As the carrier of the magnetic capturing probe, the gold-magnetic nanoparticles were coated with SPA and coupled with pcAb to construct magnetic capturing probes, then Ru(bpy)_3_^2+^-labeled phage displayed antibody was used as a specific luminescence probe, and the ECL immunosensor based on gold-magnetic nanoparticles and phage displayed antibodies was obtained. A new method for trace toxin detection was set up, and it realized detection of trace ricin levels. The LOD was 0.0001 µg/L, which was 2500-fold lower than that of conventional ELISA. The gold-magnetic nanoparticles, SPA and Ru(bpy)_3_^2+^-labeled phage displayed antibody displayed different amplifying effects in the ECL immunosensor and can decrease LOD 3-fold, 3-fold and 20-fold, respectively. The detection limit of the sensor was decreased 180 times as a result of the integrated amplifying effect of the above three components.

## 5. Conclusions

This paper proposed a new ECL immunosensor for biological trace sample detection. The immunosensor integrates the unique advantages of an ultra-highly sensitive ECL technique, a SPA-coated gold-magnetic nanoparticle-functionalized probe as magnetic capturing probe, and Ru(bpy)_3_^2+^-labeled phage displayed antibodies as luminescence probe. Such an integration idea shows good application prospects for the detection of complex biological samples that require high sensitivity, specificity and interference-resistance. The ECL immunosensor is proven well suitable for the analysis of trace levels of ricin in various environmental samples with high recovery ratio and reproducibility. The linear range of the sensor was 0.0001~200 µg/L, and the LOD was 0.0001 µg/L, which represented a 2500-fold lower one than that of conventional ELISA. The gold-magnetic nanoparticles, SPA and Ru(bpy)_3_^2+^-labeled phage displayed antibodies displayed different amplifying effects in the ECL immunosensor and can decrease LOD 3-fold, 3-fold and 20-fold compared with the ECL immunosensor without one of the three effects, respectively. The integrated amplifying effect can decrease the LOD 180 times.

## Figures and Tables

**Table 1:** Absorbance value of SPA solution before and after binding to magnetic nanoparticles (*n* = 5).

Added Amount (µg)	*A_280nm_* before	*A_280nm_* after	Binding Ratio (%)	Immobilized Amount (µg)
40	0.428 ± 0.005	0.043 ± 0.003	75.0	30
80	0.773 ± 0.007	0.106 ± 0.006	72.5	58
160	1.077 ± 0.008	0.249 ± 0.005	63.8	102
240	1.498 ± 0.007	0.530 ± 0.008	46.3	111
320	1.959 ± 0.009	0.931 ± 0.008	35.3	113
400	2.443 ± 0.008	1.404 ± 0.009	29.3	117
500	3.037 ± 0.009	1.998 ± 0.008	24.0	120

**Table 2:** Absorbance value of SPA solution before and after binding to gold-magnetic nanoparticles.

Added Amount (µg)	*A_280nm_* before	*A_280nm_* after	Binding Ratio (%)	Immobilized Amount (µg)
40	0.428 ± 0.006	0.107 ± 0.004	90.0	36
80	0.773 ± 0.008	0.213 ± 0.006	86.3	69
160	1.077 ± 0.007	0.390 ± 0.006	76.9	123
240	1.498 ± 0.008	0.800 ± 0.007	64.6	155
320	1.959 ± 0.009	1.267 ± 0.008	52.5	168
400	2.443 ± 0.008	1.720 ± 0.007	42.3	169
500	3.037 ± 0.009	2.308 ± 0.009	34.2	171

**Table 3:** Absorbance value of BSA solution before and after binding to gold-magnetic nanoparticles.

Added Amount (µg)	*A_280nm_* before	*A_280nm_* after	Binding Ratio (%)	Immobilized Amount (µg)
40	0.434 ± 0.008	0.043 ± 0.006	90.0	36
80	0.795 ± 0.009	0.099 ± 0.007	87.5	70
160	1.094 ± 0.008	0.219 ± 0.008	80.0	128
240	1.518 ± 0.009	0.411 ± 0.006	72.9	175
320	1.923 ± 0.007	0.600 ± 0.009	68.8	220
400	2.446 ± 0.009	0.824 ± 0.008	66.3	265
480	2.956 ± 0.010	1.318 ± 0.009	55.4	266
560	3.550 ± 0.010	1.864 ± 0.010	47.5	266

**Table 4:** Selective enrichment of anti-ricin phage displayed antibodies from the libraries during panning.

Round	Phage Input (PFU)	Phage Output (PFU)	Recovery Ratio (%)
1	3.3 × 10^12^	6.2 × 10^5^	1.9 × 10^−7^
2	9.6 × 10^11^	9.7 × 10^5^	1.0 × 10^−6^
3	1.4 ×10^12^	2.2 × 10^6^	1.6 × 10^−6^

Recovery ratio (%) = (No. of phage output)/(No. of phage input) × 100%.

**Table 5:** Comparison of five ECL immunosensor and ELISA (Method 1~5 see Figure 9; Method 6: conventional double-antibody sandwich ELISA.)

Detecting Scheme	Linear Range (μg/L)	Regression Equation	Correlation Coefficient (R)	Limit of Detection (μg/L)
Method 1	0.0001~200	Y = 219.42X + 748.61	0.9903	0.0001
Method 2	0.0003~200	Y = 204.83X + 643.88	0.9918	0.0003
Method 3	0.0003~200	Y = 194.17X + 613.61	0.9919	0.0003
Method 4	0.002~500	Y = 171.42X + 398.51	0.9905	0.002
Method 5	0.018~500	Y = 158.86X + 236.21	0.9913	0.018
Method 6	0.25~250	Y = 0.5415X + 0.6196	0.9945	0.25

**Table 6:** Summary of ricin detection methods.

Detection Method	Ricin Enrichment Method	Sample Matrix	LOD	Time	Reference
Sandwich-type ELISA based on microwave irradiation and heat	Antibody conjugated to 96-well plate	Food Samples	10 ppb	2 h	[37]
ELISA and Luminex fluid array assays	sdAb conjugated to 96-well plate	Buffer	1 ng/mL and 64 pg/mL	Not reported	[38]
Lateral flow devices	Antibody conjugated nitrocellulose membrane	Cosmetics	0.01 μg/mL	Not reported	[39]
Colloidal immunochromatographic assay	Antibody conjugated nitrocellulose membrane	Buffer	0.1~50 ng/mL	10 min	[40]
Electrochemiluminescence assay	Antibody conjugated to 96-well plate	Buffer	50 pg/mL	2.5 h	[41]
Magnetoelastic sensor	Antibody conjugated to sensor surface	Water, blood and serum	5 ng/mL	3.5 h	[42]
Microring resonator array	sdAb conjugated to microring resonator array	Buffer	300 pM	15 min	[43]
Surface plasmon resonance based on sdAb-QD	sdAb conjugated to SPR chip	Buffer	1 ng/mL and 0.7 ng/mL	2~6 min	[44]
Antibody-sandwich surface plasmon resonance sensor	Antibody conjugated to SPR chip	Buffer	3 ng/mL	<30 min	[45]
Liquid-crystal based sensor	Antibody conjugated to liquid crystals supported surfaces	Buffer	10 μg/mL	1–2 h	[46]
Electrochemical aptamer scaffold biosensors	Aptamer conjugated to gold electrode surface	Buffer	0.3~0.1 nM	Not reported	[47]
Nanoelectrode array biosensor based on carbon nanofiber	Antibody or aptamer conjugated to the carbon nanofibers chips	Buffer	<1 pM	4 h	[48]
DNA aptamer and Raman scattering technique	Aptamer conjugated to magnetic particles	Buffer and beverages	25 ng/mL	Not reported	[49]
Immuno-PCR	Antibody conjugated to microtitration plate	Ground beef, milk, and egg	0.01~0.1 ng/mL	Not reported	[50]
Real-time fluorescence PCR of nanoparticle-based bio-barcode	Antibody conjugated to magnetic nanoparticle	Buffer	1 fg/mL	Not reported	[51]
Nano LC–MS	Lactose-immobilized monolithic spin column	High protein solution	8 ng/mL	5 h	[52]

**Table 7:** Detection specificity of ECL immunosensor (*n* = 5).

Object	ECL Intensity (a.u.)	Relative Standard Deviation (%)
Ricin	737.8 ± 13.5	1.8
Abrin	5.2 ± 0.5	8.6
SEB	5.6 ± 0.5	9.8
BSA	5.6 ± 0.5	9.8
River water	4.8 ± 0.4	9.3
Fertilized soil	5.2 ± 0.4	8.6
Butter biscuit	5.2 ± 0.4	8.6
Whole rabbit blood	5.6± 0.5	9.8
PBS buffer	5.2 ± 0.4	8.6

**Table 8:** Determination of the simulated ricin specimens by ECL immunosensor (*n* = 4).

Sample	Added (μg/L)	Found (μg/L)	Recovery Ratio (%)	Relative Standard Deviation (%)
River water	5	4.71 ± 0.15	94.2	3.08
Fertilized soil	5	4.58 ± 0.07	91.6	1.46
Butter biscuit	5	4.55 ± 0.14	91.1	2.97
Whole rabbit blood	5	4.54 ± 0.11	90.8	2.38

**Table 9:** Determination of the simulated ricin specimens by conventional double-antibody sandwich ELISA (*n* = 4).

Sample	Added (μg/L)	Found (μg/L)	Recovery Ratio (%)	Relative Standard Deviation (%)
River water	5	4.61 ± 0.12	92.2	2.60
Fertilized soil	5	5.77 ± 0.11	115.4	1.91
Butter biscuit	5	4.55 ± 0.09	91.0	1.98
Whole rabbit blood	5	5.96 ± 0.14	119.2	2.35
